# Preferences for Religious and Spiritual Discussion in Saudi Psychiatric Care

**DOI:** 10.7759/cureus.116059

**Published:** 2026-09-10

**Authors:** Mohammed Bogari, Mohammed Maddah, Barakat Mamdouh

**Affiliations:** 1 Psychiatry, Makkah Health Cluster, Makkah, SAU; 2 Psychiatry, King Abdulaziz Hospital, Makkah, SAU

**Keywords:** cross-sectional studies, patient preference, psychiatry, religion, saudi arabia, spirituality

## Abstract

Objective: This study aimed to measure preferences of Saudi psychiatric outpatients regarding psychiatrist-initiated discussion of religion and spirituality (R/S) and to identify sociodemographic, clinical, and religiosity-related factors independently associated with these preferences.

Methods: In this cross-sectional study, 202 adult psychiatric outpatients at Al-Noor Specialist Hospital, Makkah, Saudi Arabia, completed a self-administered questionnaire assessing sociodemographic and clinical characteristics, religiosity, and preferences for R/S discussion. The primary outcome, receptiveness to R/S discussion, was modeled using Poisson regression with robust standard errors. A secondary continuous outcome was modeled using linear regression. False discovery rate correction was applied across baseline comparisons.

Results: Of 259 patients approached, 202 (78.0%) participated in the study. A total of 38.6% of participants actively preferred psychiatrist-initiated discussion of R/S, and 43.6% were open to it if clinically relevant (combined: 82.2%, 95% CI: 76.3-86.8), while 9.9% preferred no discussion and 6.4% preferred to raise the topic themselves. The prevalence estimate was sensitive to outcome definition, ranging from 38.6% to 88.6% across four prespecified definitions, whereas the religiosity association was stable across all four. When the three Duke University Religion Index (DUREL) domains were entered simultaneously, intrinsic religiosity was the only domain independently associated with the outcome (adjusted prevalence ratio (aPR): 1.12 per point, 95% CI: 1.07-1.17, p < 0.001); organizational religiosity contributed modestly (aPR: 1.05, 95% CI: 1.01-1.10, p = 0.008) and non-organizational religiosity showed no independent association (aPR: 1.01, 95% CI: 0.96-1.08, p = 0.630). Older age was associated with greater acceptability in the primary model but was attenuated on further adjustment. Sex and education were not independently associated with the outcome.

Conclusion: Most Saudi psychiatric outpatients in this single-center sample considered psychiatrist-initiated discussion of R/S acceptable, particularly those with higher intrinsic religiosity, although a substantial minority preferred no discussion or preferred to raise the topic themselves. These findings describe stated preferences and do not establish the benefit of routine spiritual assessment; they support individualized, permission-based inquiry rather than routine implementation.

## Introduction

Religion and spirituality (R/S) are increasingly recognized as relevant dimensions of psychiatric care. A systematic review of 12,327 patients found that, across the majority of studies, over half of patients considered it appropriate for a doctor to inquire about spiritual needs in at least some circumstances, with a median endorsement of 70.5% [1]. Studies have consistently linked religious and spiritual involvement to better mental health outcomes [2], and this convergence between patient interest and clinical relevance has been formally acknowledged by the World Psychiatric Association, whose position statement affirms that spirituality and religion should be considered a routine part of psychiatric assessment [3]. The inclusion of a religious or spiritual problem category in the Diagnostic and Statistical Manual of Mental Disorders, 4th edition (DSM-IV) further reflected a shift toward recognizing such experiences as clinically meaningful rather than inherently pathological [4]. Recent work continues to support integrating R/S into psychiatric outpatient care in Western settings, reinforcing that this is an evolving standard of person-centered practice rather than a marginal concern [5].

This international pattern takes on particular significance in the Eastern Mediterranean and wider Arab region, where religion is deeply embedded in daily life, personal identity, and the interpretation of illness itself [6]. Traditional and faith healers remain a common, often first-line, resource for individuals experiencing psychiatric symptoms across Arab countries, a pattern consolidated in a recent systematic review of the Arab-world literature on faith healing and psychiatric illness [7]. Large multinational surveys across the region have similarly shown that religiosity shapes both stigma toward mental illness and attitudes toward help-seeking [8], and a systematic review of help-seeking experiences among Arab populations worldwide identified religious belief as a recurring theme shaping whether and how individuals engage with formal mental health services [9]. Together, this literature underscores both the centrality of R/S to how patients in this region experience psychiatric illness and the corresponding importance of clinicians being equipped to engage with these beliefs directly.

Saudi Arabia exemplifies this context: a cross-sectional study among Saudi psychiatric outpatients found that a substantial proportion of individuals who visited faith healers had diagnosable, previously untreated psychiatric disorders [10], and a recent national survey of Saudi psychiatry residents found that although most considered it appropriate to inquire about patients' spiritual concerns, nearly all had received no formal training in doing so and were markedly less comfortable raising R/S than their North American counterparts [11] - a discrepancy suggesting a gap between what patients may value and what clinicians feel equipped to address. Notably, this literature has focused almost exclusively on clinicians' attitudes; to our knowledge, no published study has directly examined Saudi psychiatric patients' own preferences regarding clinician-initiated R/S discussion, or the factors associated with these preferences.

The primary objective of this study was to estimate the proportion of psychiatric outpatients at Al-Noor Specialist Hospital, Makkah, who consider psychiatrist-initiated discussion of religion or spirituality acceptable, reporting the full distribution of preferences rather than a single composite. Secondary objectives were to examine associations between this preference and religiosity, measured across the three domains of the five-item Duke University Religion Index (DUREL) developed by Koenig and Büssing [12] (organizational religious activity, non-organizational religious activity, and intrinsic religiosity) and sociodemographic characteristics. Associations with marital status, psychiatric diagnosis, and prior experience of religious or spiritual discussion were examined as exploratory objectives. Given the cross-sectional design, all associations are reported as associational rather than causal.

## Materials and methods

This study is reported in accordance with the STROBE (Strengthening the Reporting of Observational Studies in Epidemiology) guidelines for cross-sectional studies [13].

Study design and setting

This was a cross-sectional, descriptive-analytical study using a self-administered, anonymous questionnaire, conducted from April 1, 2026, to June 30, 2026, in the psychiatric outpatient clinics of Al-Noor Specialist Hospital, Makkah, Saudi Arabia, a tertiary referral center serving a diverse adult psychiatric population.

Participants

Participants were eligible if they were adult patients diagnosed with a psychiatric disorder, including mood, anxiety, psychotic, or other psychiatric disorders, were able to read and understand Arabic, and provided informed consent. Patients were excluded if they were experiencing an acute psychotic or manic episode associated with impaired insight or comprehension, or had cognitive impairment that precluded completion of the questionnaire.

Psychiatric diagnoses were assigned by the treating psychiatrist according to the Diagnostic and Statistical Manual of Mental Disorders, Fifth Edition (DSM-5) criteria as part of routine clinical assessment and were recorded from the clinical record rather than reported by the participant [14]. Where more than one diagnosis was present, the primary diagnosis recorded by the treating psychiatrist was used; comorbid diagnoses were not analyzed separately. Diagnoses outside the listed categories, together with three respondents for whom no diagnosis was recorded, were combined into a single "Other" category (n = 11). No diagnostic data were missing.

Biological sex (male/female) was recorded as reported by participants. Gender identity was not assessed separately because the demographic variable of interest in this study was biological sex as a sociodemographic characteristic rather than gender identity.

Sampling

A non-probability consecutive sampling technique was used. Recruitment took place in the psychiatric outpatient clinics from Sunday to Thursday, 08:00-16:00, throughout the data-collection period. Patients were approached at routine appointments, which included both first assessments and follow-up visits. Eligible patients attending during these periods were invited consecutively by two psychiatry consultants and one psychiatry resident who were members of the research team, after the study purpose was explained and informed consent obtained. The questionnaire was completed within the clinic after the patient's consultation had concluded, in digital form only; no paper questionnaires were used. As recruitment was face-to-face, these team members necessarily knew which patients agreed to participate; however, responses were submitted anonymously to an online form, no identifying information was collected, and no response could be linked to an individual patient. The online form did not restrict repeat submission from the same device, so duplicate entries cannot be excluded.

Sample size was calculated using Cochran's formula for proportion estimation with an expected prevalence of 50%, 95% confidence level, and 5% margin of error, yielding a target of 384. Recruitment was bounded by a prespecified three-month data-collection window determined by the clinical rotation during which the study was conducted, and enrolment ended when this window closed rather than on reaching the calculated target. Of 259 patients approached, 57 declined, and 202 completed the questionnaire, giving a response rate of 78.0%. Reasons for refusal were not systematically recorded.

Instruments

Participants completed a self-administered, structured questionnaire in Arabic, delivered as an online form and requiring approximately 10 minutes to complete. The questionnaire comprised three parts: (1) a demographic and clinical data sheet recording sex, age, marital status, educational level, self-rated level of religious or spiritual commitment, psychiatric diagnosis category, duration of psychiatric follow-up, and prior experience of discussing R/S with a psychiatrist; (2) the Arabic version of the five-item DUREL; and (3) three study-specific preference items assessing desire for psychiatrist-initiated R/S discussion, agreement to discuss R/S during the visit if clinically relevant (five-point Likert item), and preferred timing for such discussion. Study-specific questions were added to the DUREL questionnaire to address the variables of interest, and the complete questionnaire, including all response options, is provided in the Appendices.

The Duke University Religion Index (DUREL)

The DUREL comprises three domains - organizational religiosity and non-organizational religiosity, each a single item rated on a six-point frequency scale, and intrinsic religiosity, three items on a five-point agreement scale - together with an optionally calculated total score. Organizational and non-organizational subscale scores range from 1 to 6 each, the intrinsic subscale scores range from 3 to 15, and the total score ranges from 5 to 27, with higher scores indicating greater religiosity. The DUREL was translated into Arabic by the authors, who are native Arabic speakers, from the original English instrument, as published by Koenig and Büssing [12]. Following the developers' explicit guidance for non-Christian samples, attendance at church was rendered as congregational prayer at the mosque or other religious gatherings, and private Bible study as supplication, remembrance, individual prayer, or reading of religious texts. Independent back-translation was not performed, and no formal cross-cultural adaptation procedure was undertaken. Permission was not separately sought from the instrument developers. To our knowledge, no formally validated Arabic version of the DUREL has been published; the version used here should therefore be regarded as a study-specific forward translation.

Development of the Preference Items

The three preference items were developed by the authors for this study, drawing on the content domains covered in previously published surveys of patient preferences regarding spiritual inquiry. Draft items were reviewed for face and content validity by the three authors together with four other psychiatrists in the department of psychiatry. No patient pilot testing was conducted before use, and the items did not undergo formal psychometric evaluation; no factor analysis or test-retest assessment was performed. Outcome coding rules were specified before analysis. An additional item asking reasons for not wishing to discuss R/S was displayed to all respondents except those selecting "yes, I would prefer that," and was therefore answered by 125 respondents, including those who indicated only conditional acceptance; reasons are consequently reported separately for the 36 respondents classified as not accepting clinician-initiated inquiry.

Statistical analysis

Outcomes

The five-category preference item is reported in full as the principal descriptive result, with Wilson 95% confidence intervals. For regression analysis, a binary outcome - acceptability of psychiatrist-initiated inquiry - was defined a priori by combining respondents who indicated they would prefer such discussion with those who would be open to it if clinically relevant, versus those who preferred no discussion, preferred to initiate the topic themselves, or were unsure. This composite deliberately combines active preference with conditional acceptance, and the two components are reported separately. As the classification of respondents preferring patient-initiated discussion is open to question, three alternative definitions were analyzed as prespecified sensitivity analyses: a broader definition, in which preference for patient-initiated discussion was classified as acceptable; a strict definition, restricted to active preference for clinician-initiated discussion; and a complete-case definition, in which unsure responses were excluded rather than classified as not acceptable. The secondary outcome was the five-point Likert item assessing agreement to discuss R/S during the visit if clinically relevant.

Descriptive Comparisons

Categorical variables were summarized as frequencies and percentages and compared using chi-square tests, with p-values obtained by Monte Carlo simulation with 10,000 replicates to accommodate sparse cells, and bias-corrected Cramér's V as the effect size. Continuous variables were summarized as medians with interquartile ranges and compared using the Wilcoxon rank-sum test following confirmation of non-normality (Anderson-Darling test), with rank-biserial correlation as the effect size. The Benjamini-Hochberg false discovery rate (FDR) correction was applied across the family of 11 baseline comparisons presented in the table showing baseline characteristics, and both raw and adjusted p-values are reported for every comparison. As the DUREL total score is a deterministic function of its component scores, only the three domains were included in this family; the total score is presented descriptively.

Primary Model

The primary outcome was modeled using Poisson regression with a log link and robust (HC3) standard errors, yielding prevalence ratios (PRs) rather than odds ratios, appropriate given the outcome's high prevalence (>10%) in this cross-sectional design. Both crude and adjusted models were estimated. The adjusted model included sex, age group, and education, selected a priori as the adjustment set on the basis of the variable map. The target estimand is the total association between religiosity and acceptability, expressed as an adjusted prevalence ratio (aPR); the analysis is associational, and aPRs are not intended to support causal interpretation. Prior experience of R/S discussion was treated as a possible mediator and deliberately left unadjusted in the primary model, since conditioning on it would remove part of the association of interest. Psychiatric diagnosis and marital status could not be confidently assigned a role in a cross-sectional design, since each may plausibly act as a confounder, as a consequence of the exposure, or as a common effect; they were therefore restricted to a separate exploratory model. No variable in the map is a collider on the exposure-outcome path.

Exposure Specification

In line with the developers' scoring guidance, which advises against summing the subscales into a total score, the exposure was modeled primarily as the three DUREL domains entered in separate models. A secondary model entered all three domains simultaneously; the developers caution that this may introduce collinearity, and variance inflation factors were therefore examined. The DUREL total score is reported only as a secondary summary index. For descriptive display, religiosity tertiles were defined by the sample quantiles of the total score (low, 5-21; moderate, 22-24; high, 25-27); group sizes are unequal because the score is discrete and tied values were kept within the same tertile.

Exploratory Model

An exploratory model additionally included psychiatric diagnosis and prior R/S discussion experience. To avoid overfitting, diagnosis was collapsed into four clinically coherent groups (anxiety/obsessive-compulsive/trauma-related, mood, psychotic, and personality/other) and age into three groups. Marital status was omitted because it is near-collinear with age in this sample. The model estimated nine coefficients against 36 observations in the limiting outcome class, giving an events-per-variable value of 4.0. All estimates from this model are exploratory and hypothesis-generating.

Secondary Outcome

The secondary outcome was modeled using proportional-odds ordinal logistic regression with the same adjustment set, which is presented as the primary analysis of this outcome. The proportional odds assumption was assessed by fitting separate binary logistic models at each cut-point. Multiple linear regression is retained as a secondary presentation, with residual diagnostics (Jarque-Bera and Breusch-Pagan tests) and the distribution of the item reported.

Additional Analyses

As a post-hoc check on the study-specific preference items, the association between the five-category preference item and the independently worded Likert agreement item was examined using Spearman's rank correlation. Sensitivity analyses included exclusion of one record from each of two pairs with identical response patterns, and a wave analysis comparing the first and last quartiles of respondents by submission timestamp to assess potential response-order bias.

Software and Reporting

All analyses were conducted in R version 4.3.5 (R Foundation for Statistical Computing, Vienna, Austria). No missing data were present for the primary outcome, DUREL items, or sociodemographic and clinical covariates. Statistical significance was set at α < 0.05 (two-tailed). P-values are reported to three decimal places, or as p < 0.001 where smaller. Effect sizes with confidence intervals are emphasized throughout in preference to p-values. The de-identified dataset and analysis code are available from the corresponding author on reasonable request.

Ethical considerations

Ethical approval was obtained from the Makkah Health Cluster Institutional Review Board (approval number: H-02-K-076-1225-1509). The study was conducted in accordance with the Declaration of Helsinki. Informed consent was obtained from all participants prior to enrollment. Participant confidentiality was maintained throughout; no identifying information was collected, and the questionnaire was completed anonymously.

## Results

Of 259 patients approached, 57 declined, and 202 completed the questionnaire, giving a response rate of 78.0%. No data were missing for the primary outcome, the DUREL items, or any sociodemographic or clinical covariate.

The three-item intrinsic religiosity subscale showed good internal consistency (Cronbach's α = 0.83). Internal consistency cannot be computed for the organizational and non-organizational domains, each of which is a single item. For completeness, α across all five items was 0.71, and inter-domain Spearman correlations were modest (organizational-non-organizational: 0.26; organizational-intrinsic: 0.24; non-organizational-intrinsic: 0.41), indicating that the three domains are empirically as well as conceptually distinct.

Under the prespecified primary definition, 166 of 202 respondents (82.2%, 95% CI: 76.3-86.8) were classified as finding psychiatrist-initiated inquiry acceptable and 36 (17.8%) were not. Respondents who did and did not find inquiry acceptable were similar by sex (91 of 166 (54.8%) versus 18 of 36 (50.0%) female; raw p = 0.712, Cramér's V = 0.000) but differed by age group (raw p < 0.001, V = 0.335), marital status (raw p < 0.001, V = 0.272), psychiatric diagnosis (raw p < 0.001, V = 0.432), self-rated religious commitment (raw p < 0.001, V = 0.492), and previous experience of discussing R/S with a psychiatrist (raw p < 0.001, V = 0.366). All of these survived FDR correction. Education showed a weaker association that did not survive correction (raw p = 0.043, FDR-adjusted p = 0.053, V = 0.169), and duration of follow-up was not associated with the outcome (raw p = 0.125, FDR-adjusted p = 0.137, V = 0.109). All three DUREL domain scores were higher among respondents who found inquiry acceptable (raw and FDR-adjusted p < 0.001 for each): organizational religiosity (acceptable: median = 4, Q1-Q3 = 2-6; non-acceptable: median = 2.5, Q1-Q3 = 2.0-4.0; rank-biserial r = −0.431); non-organizational religiosity (acceptable: median = 6, Q1-Q3 = 5-6; non-acceptable: median = 4.0, Q1-Q3 = 2.8-6.0; r = −0.415); and intrinsic religiosity (acceptable: median = 14, Q1-Q3 = 12-15; non-acceptable: median = 10.0, Q1-Q3 = 9.0-12.0; r = −0.680) (Table 1).

**Table 1:** Baseline characteristics by acceptability of psychiatrist-initiated religion/spirituality discussion (N = 202). Values are n (%) unless stated. "Acceptable" denotes respondents who preferred psychiatrist-initiated discussion of religion/spirituality or were open to it if clinically relevant. Categorical comparisons used Monte Carlo exact chi-square tests with 10,000 replicates; continuous comparisons used the Wilcoxon rank-sum test. The Benjamini-Hochberg false discovery rate correction was applied across the family of 11 comparisons shown. Effect sizes are bias-corrected Cramér’s V for categorical variables and rank-biserial r for continuous variables. The DUREL total score is presented descriptively and was excluded from the correction family because it is a deterministic function of its three domains. R/S = religion and spirituality; DUREL = Duke University Religion Index; FDR = false discovery rate.

Characteristic	Overall (N = 202)	Not acceptable (n = 36)	Acceptable (n = 166)
Sex (raw p = 0.712; FDR p = 0.712; V = 0.000)			
Female	109 (54.0)	18 (50.0)	91 (54.8)
Male	93 (46.0)	18 (50.0)	75 (45.2)
Age group, years (raw p < 0.001; FDR p < 0.001; V = 0.335)			
18-25	44 (21.8)	19 (52.8)	25 (15.1)
26-35	53 (26.2)	8 (22.2)	45 (27.1)
36-45	49 (24.3)	6 (16.7)	43 (25.9)
46-55	34 (16.8)	2 (5.6)	32 (19.3)
>55	22 (10.9)	1 (2.8)	21 (12.7)
Marital status (raw p < 0.001; FDR p < 0.001; V = 0.272)			
Single	84 (41.6)	26 (72.2)	58 (34.9)
Married	95 (47.0)	8 (22.2)	87 (52.4)
Divorced/widowed	23 (11.4)	2 (5.6)	21 (12.7)
Education (raw p = 0.043; FDR p = 0.053; V = 0.169)			
Less than secondary	17 (8.4)	0 (0.0)	17 (10.2)
Secondary	67 (33.2)	12 (33.3)	55 (33.1)
Diploma	15 (7.4)	1 (2.8)	14 (8.4)
Bachelor	94 (46.5)	23 (63.9)	71 (42.8)
Postgraduate	9 (4.5)	0 (0.0)	9 (5.4)
Psychiatric diagnosis (raw p < 0.001; FDR p < 0.001; V = 0.432)			
Anxiety disorder	53 (26.2)	6 (16.7)	47 (28.3)
Depression	62 (30.7)	11 (30.6)	51 (30.7)
Bipolar disorder	23 (11.4)	2 (5.6)	21 (12.7)
Borderline personality disorder	18 (8.9)	13 (36.1)	5 (3.0)
Obsessive-compulsive disorder	15 (7.4)	0 (0.0)	15 (9.0)
Schizophrenia/psychosis	13 (6.4)	1 (2.8)	12 (7.2)
Post-traumatic stress disorder	7 (3.5)	2 (5.6)	5 (3.0)
Other	11 (5.4)	1 (2.8)	10 (6.0)
Duration of psychiatric follow-up (raw p = 0.125; FDR p = 0.137; V = 0.109)			
<6 months	54 (26.7)	8 (22.2)	46 (27.7)
6-12 months	37 (18.3)	11 (30.6)	26 (15.7)
>1 year	111 (55.0)	17 (47.2)	94 (56.6)
Self-rated religious commitment (raw p < 0.001; FDR p < 0.001; V = 0.492)			
Not religious	7 (3.5)	3 (8.3)	4 (2.4)
Mildly religious	48 (23.8)	24 (66.7)	24 (14.5)
Moderately religious	114 (56.4)	9 (25.0)	105 (63.3)
Highly religious	33 (16.3)	0 (0.0)	33 (19.9)
Previous R/S discussion with a psychiatrist (raw p < 0.001; FDR p < 0.001; V = 0.366)			
Never discussed	143 (70.8)	25 (69.4)	118 (71.1)
Discussed — helpful	43 (21.3)	1 (2.8)	42 (25.3)
Discussed — not helpful	16 (7.9)	10 (27.8)	6 (3.6)
DUREL scores, median (Q1, Q3)			
Organizational religiosity (1-6) — raw p < 0.001; FDR p < 0.001; r = −0.431	4 (2, 6)	2.5 (2.0, 4.0)	4 (2, 6)
Non-organizational religiosity (1–6) — raw p < 0.001; FDR p < 0.001; r = −0.415	6 (4, 6)	4.0 (2.8, 6.0)	6 (5, 6)
Intrinsic religiosity (3–15) — raw p < 0.001; FDR p < 0.001; r = −0.680	13 (12, 15)	10.0 (9.0, 12.0)	14 (12, 15)
Total score (5–27) — descriptive only	23 (20, 25)	17.0 (14.8, 20.2)	23 (21, 25)

The covariate structure informing the primary model is shown in Figure 1.

**Figure 1:**
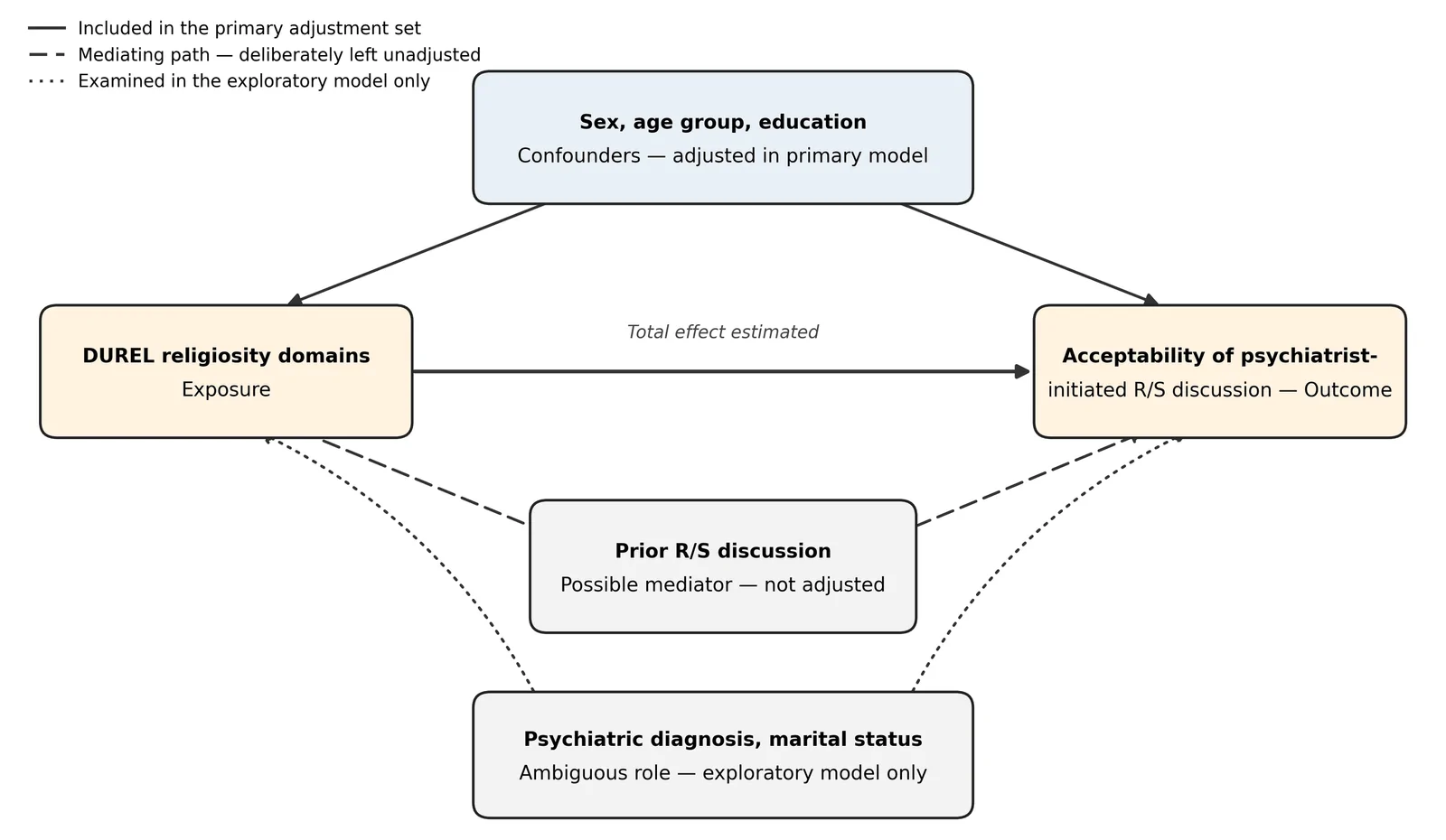
Variable map informing covariate selection. Directed graph showing the assumed relationships among the exposure, the outcome, and candidate covariates. Solid arrows indicate variables included in the primary adjustment set. The dashed path indicates prior experience of religious or spiritual (R/S) discussion, treated as a possible mediator and deliberately left unadjusted. Dotted paths indicate psychiatric diagnosis and marital status, whose roles could not be confidently assigned in a cross-sectional design and which were examined in a separate exploratory model. The graph represents assumptions rather than established causal structure; all reported estimates are associational. DUREL = Duke University Religion Index.

The proportion of respondents actively preferring clinician-initiated discussion rose across religiosity tertiles, from 12 of 73 (16.4%) in the lowest tertile to 29 of 72 (40.3%) in the middle and 37 of 57 (64.9%) in the highest (Figure 2).

**Figure 2:**
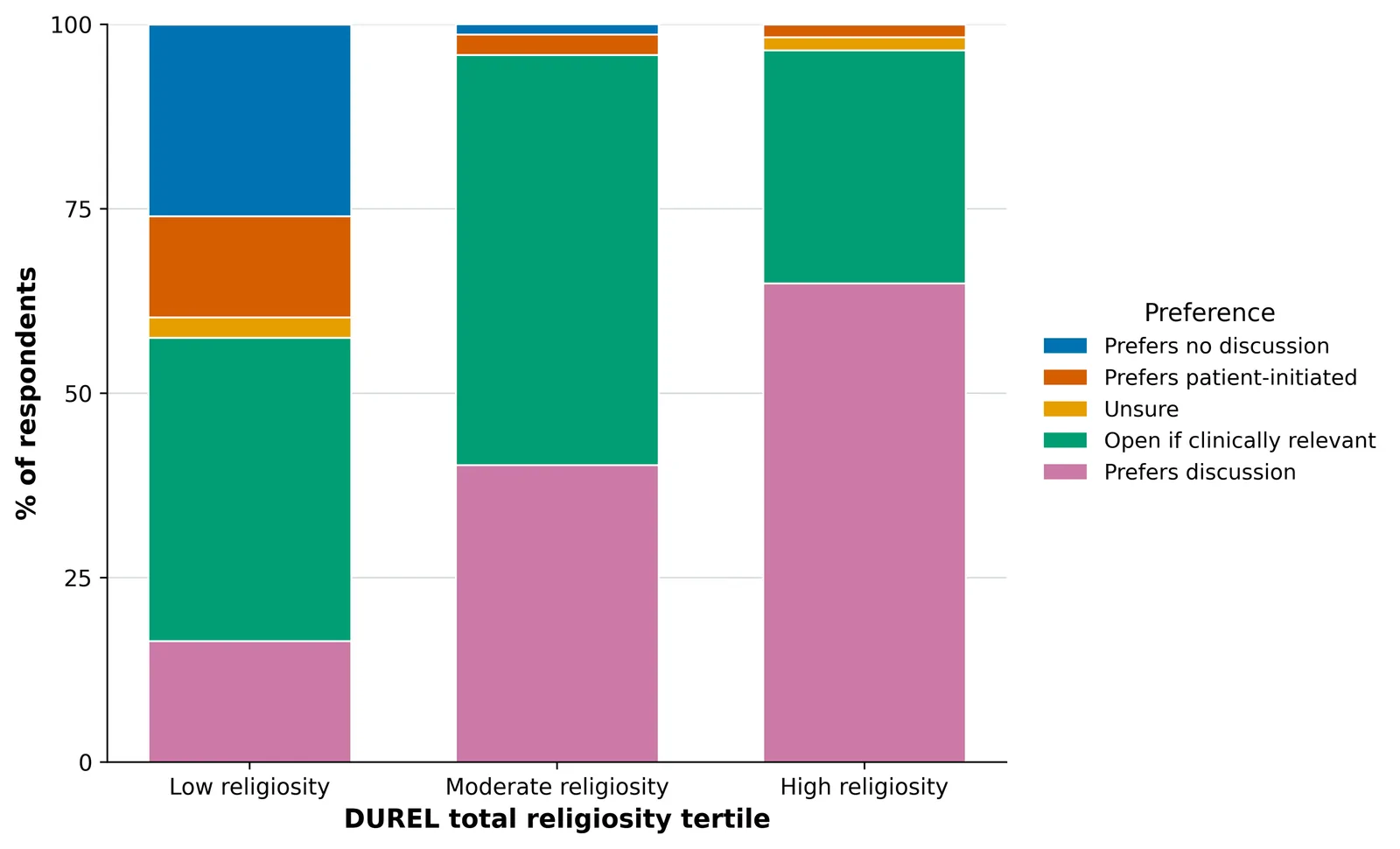
Preference for religion/spirituality (R/S) discussion by DUREL religiosity tertile. Stacked bars show the distribution of the five-category preference item within tertiles of the DUREL total religiosity score. Tertiles were defined by the sample quantiles of the total score: low, 5-21 (n = 73); moderate, 22-24 (n = 72); high, 25-27 (n = 57). Group sizes are unequal because the score is discrete, and tied values were kept within the same tertile. DUREL = Duke University Religion Index.

Seventy-eight respondents (38.6%, 95% CI: 32.2-45.5) would prefer their psychiatrist to raise religion or spirituality, and a further 88 (43.6%, 95% CI: 36.9-50.5) would not mind if it were clinically relevant. Thirteen (6.4%) would prefer to raise the topic themselves, three (1.5%) were unsure, and 20 (9.9%) would prefer no discussion. The prevalence estimate was markedly sensitive to how the outcome was defined: classifying preference for patient-initiated discussion as acceptable raised it to 88.6% (95% CI: 83.5-92.3), restricting the outcome to active preference for clinician-initiated discussion lowered it to 38.6% (95% CI: 32.2-45.5), and excluding unsure responses gave 83.4% (95% CI: 77.6-87.9). The association with religiosity was materially unchanged across all four definitions (aPR: 1.05-1.10 per DUREL point, all p ≤ 0.006) (Table 2).

**Table 2:** Distribution of the preference outcome and sensitivity of the prevalence estimate to outcome definition (N = 202). Upper panel: Wilson 95% confidence intervals. Lower panel: the primary definition was specified before analysis. The three alternatives are prespecified sensitivity analyses addressing how respondents who preferred to raise the topic themselves and unsure responses are classified. Prevalence ratios are from Poisson regression with a log link and robust (HC3) standard errors, adjusted for sex, age group, and education; aPR is per one-point increase in DUREL total score. The prevalence estimate varies substantially with outcome definition (38.6% to 88.6%), whereas the religiosity association is stable across all four. DUREL = Duke University Religion Index; CI = confidence interval; aPR = adjusted prevalence ratio.

Response option	n	%	95% CI	
Would prefer the psychiatrist to raise it	78	38.6	32.2-45.5	
Would not mind if clinically relevant	88	43.6	36.9-50.5	
Would prefer to raise the topic myself	13	6.4	3.8-10.7	
Unsure/no fixed view	3	1.5	0.5-4.3	
Would prefer no discussion	20	9.9	6.5-14.8	
Outcome definition	Categories counted as acceptable	Prevalence % (95% CI)	DUREL aPR (95% CI)	p
Primary definition	Would prefer the psychiatrist to raise it, or would not mind if clinically relevant	82.2 (76.3-86.8)	1.07 (1.04-1.10)	<0.001
Broader definition	As above, plus would prefer to raise the topic myself	88.6 (83.5-92.3)	1.05 (1.03-1.08)	<0.001
Strict definition	Would prefer the psychiatrist to raise it only	38.6 (32.2-45.5)	1.10 (1.03-1.19)	0.006
Complete-case definition	Primary definition with unsure responses excluded (n = 199)	83.4 (77.6-87.9)	1.07 (1.04-1.10)	<0.001

As a post-hoc check on the study-specific preference items, the five-category preference item correlated moderately strongly with the independently worded five-point Likert agreement item (Spearman ρ = 0.614, p < 0.001), and preference varied markedly with previous experience of R/S discussion: 42 of 43 respondents (97.7%) who had found a previous discussion helpful were classified as finding inquiry acceptable, compared with six of 16 (37.5%) who had not (p < 0.001). These observations provide limited supportive evidence of construct validity but do not substitute for formal validation.

Among the 36 respondents who did not find clinician-initiated inquiry acceptable, the most frequently endorsed reasons were a wish to keep R/S private (20 respondents, 55.6%) and fear of being misunderstood or judged (15, 41.7%), followed by concern that discussion could affect the objectivity of treatment (12, 33.3%), seeing no connection between religion and psychiatric treatment (11, 30.6%), the view that a psychiatrist is not the appropriate professional for such discussion (10, 27.8%), holding different beliefs and preferring not to discuss them (6, 16.7%), and a previous uncomfortable experience of such discussion (6, 16.7%). Respondents could endorse more than one reason.

When asked when discussion of religion or spirituality would be appropriate, 63 respondents (31.2%) answered at any time, 42 (20.8%) only if related to their psychiatric problem, 37 (18.3%) only if they requested it themselves, 31 (15.3%) at the first visit, and 29 (14.4%) only after a therapeutic relationship had been established. Of the 37 who considered discussion appropriate only on their own request, 27 (73.0%) were classified as not accepting clinician-initiated inquiry.

The distribution of DUREL total scores was shifted upward among respondents who found inquiry acceptable (median = 23, Q1-Q3 = 21-25) compared with those who did not (median = 17.0, Q1-Q3 = 14.8-20.2) (Figure 3).

**Figure 3:**
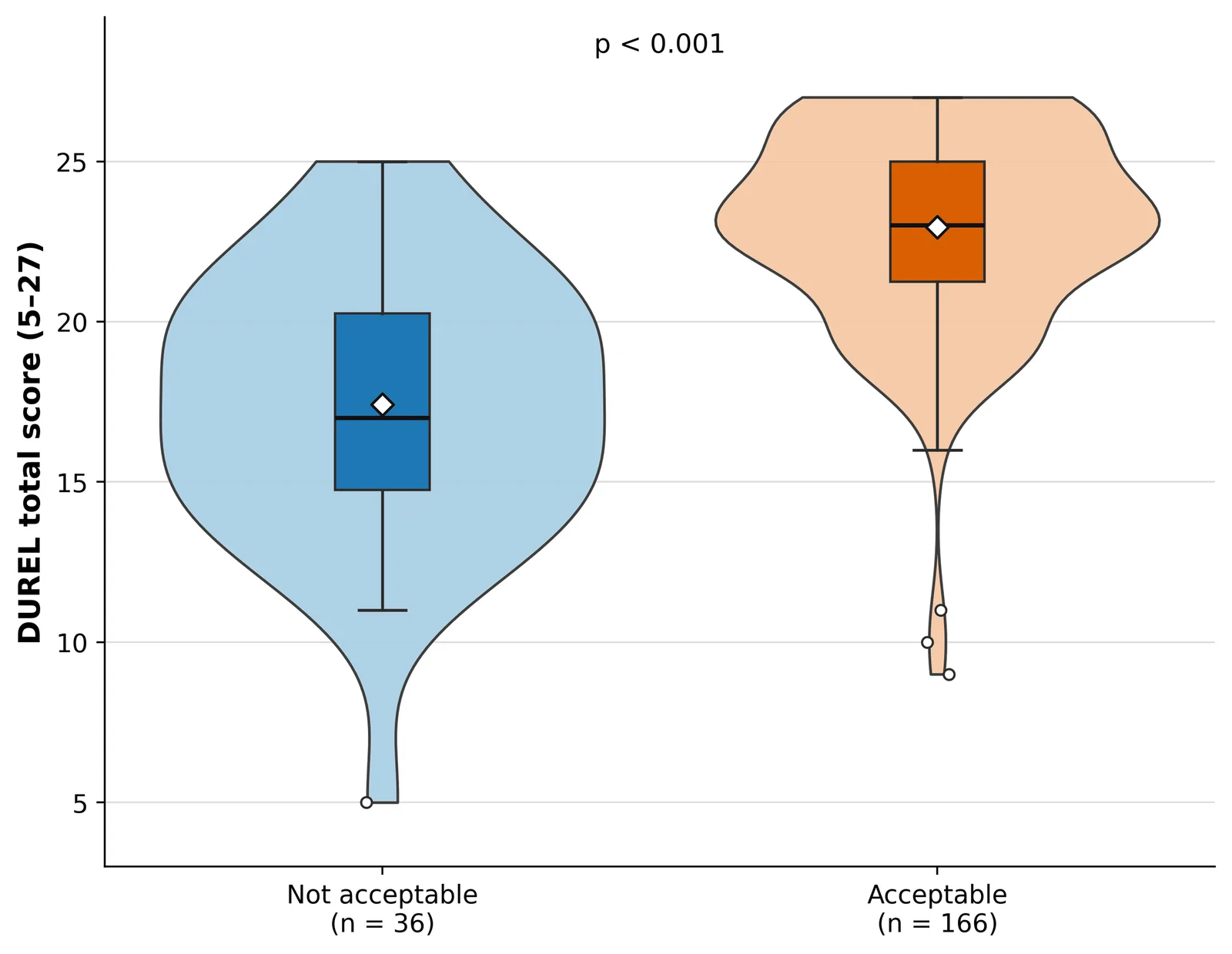
Distribution of DUREL total religiosity score by acceptability of psychiatrist-initiated religion/spirituality (R/S) discussion. Violin plots show the distribution of total DUREL scores; boxes show the interquartile range, horizontal lines within boxes show the median, white diamonds show the mean, and open circles show values beyond 1.5 times the interquartile range. Blue denotes respondents who did not find clinician-initiated discussion acceptable (n = 36); orange denotes those who did (n = 166). Wilcoxon rank-sum test, p < 0.001. DUREL = Duke University Religion Index.

Each DUREL domain was independently associated with acceptability when modeled separately: organizational religiosity (aPR: 1.09 per point, 95% CI: 1.05-1.15, p < 0.001), non-organizational religiosity (aPR: 1.10, 95% CI: 1.03-1.18, p = 0.004), and intrinsic religiosity (aPR: 1.13, 95% CI: 1.08-1.18, p < 0.001). When all three domains were entered simultaneously, the association was carried almost entirely by intrinsic religiosity (aPR: 1.12, 95% CI: 1.07-1.17, p < 0.001), with a small residual contribution from organizational religiosity (aPR: 1.05, 95% CI: 1.01-1.10, p = 0.008) and no independent contribution from non-organizational religiosity (aPR: 1.01, 95% CI: 0.96-1.08, p = 0.630). The DUREL total score, retained as a secondary summary index, gave an aPR of 1.07 per point (95% CI: 1.04-1.10, p < 0.001), essentially unchanged from the crude estimate (PR: 1.07, 95% CI: 1.05-1.10). Relative to the 18-25 year reference group, all older age groups showed a higher adjusted prevalence of acceptability: 26-35 years (aPR: 1.46, 95% CI: 1.13-1.88, p = 0.003), 36-45 years (aPR: 1.43, 95% CI: 1.12-1.83, p = 0.004), 46-55 years (aPR: 1.41, 95% CI: 1.11-1.81, p = 0.006), and over 55 years (aPR: 1.33, 95% CI: 1.04-1.70, p = 0.025). Sex was not independently associated with the outcome (aPR: 0.91, 95% CI: 0.80-1.02, p = 0.118). Education estimates were unstable because two strata contained no respondents in the non-acceptable group (less than secondary, 0 of 17; postgraduate, 0 of 9), producing complete separation against the reference category, and are not interpreted (Table 3).

**Table 3:** Predictors of acceptability of psychiatrist-initiated religion/spirituality discussion (Poisson regression with robust standard errors, N = 202). Upper panel: crude estimates are from single-predictor models; adjusted estimates are from one model containing all listed variables. The crude estimate for postgraduate education is not estimable because all nine respondents in that stratum found the inquiry acceptable, producing complete separation. All models were adjusted for sex, age group, and education where applicable. In the lower panel, "separate models" denotes one model per domain, as recommended by the instrument developers, who advise against summing the subscales into a total score; "mutually adjusted" denotes a single model containing all three domains. The developers caution that the latter may introduce collinearity, but variance inflation factors were 1.13, 1.34, and 1.33 for the organizational, non-organizational, and intrinsic domains, respectively, indicating this was not a material concern in these data. Education estimates are unstable because two strata contained no non-acceptable respondents (less than secondary, 0 of 17; postgraduate, 0 of 9) and are not interpreted. DUREL = Duke University Religion Index; PR = prevalence ratio; aPR = adjusted prevalence ratio.

Characteristic	Crude PR (95% CI)	p	Adjusted PR (95% CI)	p
DUREL total score, per point	1.07 (1.05-1.10)	<0.001	1.07 (1.04-1.10)	<0.001
Sex: male vs. female	0.97 (0.85-1.10)	0.602	0.91 (0.80-1.02)	0.118
Age 26-35 vs. 18-25	1.49 (1.13-1.98)	0.005	1.46 (1.13-1.88)	0.003
Age 36-45 vs. 18-25	1.54 (1.17-2.04)	0.002	1.43 (1.12-1.83)	0.004
Age 46-55 vs. 18-25	1.66 (1.26-2.17)	<0.001	1.41 (1.11-1.81)	0.006
Age >55 vs. 18-25	1.68 (1.28-2.21)	<0.001	1.33 (1.04-1.70)	0.025
Education: secondary vs. less than secondary	0.82 (0.73-0.92)	0.001	1.03 (0.90-1.18)	0.668
Education: diploma vs. less than secondary	0.93 (0.82-1.07)	0.317	1.25 (1.00-1.56)	0.048
Education: bachelor vs. less than secondary	0.76 (0.67-0.85)	<0.001	0.90 (0.78-1.05)	0.169
Education: postgraduate vs. less than secondary	Not estimable	—	1.09 (0.88-1.35)	0.434
DUREL exposure	Separate models aPR (95% CI)	p	Mutually adjusted aPR (95% CI)	p
Organizational religiosity, per point	1.09 (1.05-1.15)	<0.001	1.05 (1.01-1.10)	0.008
Non-organizational religiosity, per point	1.10 (1.03-1.18)	0.004	1.01 (0.96-1.08)	0.63
Intrinsic religiosity, per point	1.13 (1.08-1.18)	<0.001	1.12 (1.07-1.17)	<0.001
Total score, per point (secondary index)	1.07 (1.04-1.10)	<0.001	—	—

An exploratory model additionally including psychiatric diagnosis and prior R/S discussion experience was fitted with diagnosis collapsed to four groups and age to three, giving nine estimated coefficients against 36 observations in the limiting outcome class (events per variable: 4.0). The association between DUREL total score and acceptability was attenuated but persisted (PR: 1.06, 95% CI: 1.03-1.09, p < 0.001). The combined personality and other diagnostic category showed lower acceptability relative to anxiety, obsessive-compulsive, and trauma-related disorders (PR: 0.69, 95% CI: 0.51-0.93, p = 0.014); among the 18 respondents with borderline personality disorder, five (27.8%) found inquiry acceptable. The age association was attenuated but directionally preserved (26-45 years, PR: 1.29, 95% CI: 1.02-1.62, p = 0.030; over 45 years, PR: 1.21, 95% CI: 0.96-1.54, p = 0.108). All estimates from this model derive from small subgroups and are hypothesis-generating only. The secondary outcome showed a marked ceiling effect: 88 respondents (43.6%) selected the highest category and 148 (73.3%) selected one of the top two, while three (1.5%) selected the lowest. Residuals from the linear model departed from normality (Jarque-Bera p < 0.001), although homoscedasticity was acceptable (Breusch-Pagan p = 0.246). In the proportional-odds ordinal logistic model, a higher DUREL total score was associated with greater agreement (odds ratio: 1.35 per point, 95% CI: 1.24-1.47, p < 0.001), as was age above 25 years. The proportional-odds assumption was supported at the three well-populated cut-points, at which the DUREL log-odds coefficient was stable (0.329, 0.288, 0.345); the lowest cut-point was too sparse to estimate reliably. Education estimates were again unstable owing to complete separation and are not interpreted. The linear model gave concordant results (β = 0.121, 95% CI: 0.092-0.151, p < 0.001; adjusted R² = 0.394) (Table 4).

**Table 4:** Exploratory clinical predictors and the secondary outcome (N = 202). Upper panel: Poisson regression with robust (HC3) standard errors; nine estimated coefficients against 36 observations in the limiting outcome class (events per variable: 4.0). Diagnosis was collapsed to four groups and age to three to avoid the overfitting present in the previous model; marital status was omitted because it is near-collinear with age in this sample (43 of 44 respondents aged 18-25 years were single; 46 of 47 aged over 45 years were married). All estimates in the upper panel are exploratory and hypothesis-generating. Lower panel: proportional-odds ordinal logistic regression is the primary analysis of this outcome, with the linear model retained for comparison; both are adjusted for sex, age group, and education. The item shows a marked ceiling effect (43.6% at the highest category; 73.3% in the top two), and linear-model residuals departed from normality (Jarque–Bera p < 0.001), although homoscedasticity was acceptable (Breusch-Pagan p = 0.246). The proportional-odds assumption was supported at the three well-populated cut-points, where the DUREL log-odds coefficient was stable (0.329, 0.288, 0.345); the lowest cut-point (n = 3 below it) was too sparse to estimate reliably. Education estimates are omitted from the lower panel because complete separation renders them uninterpretable. DUREL = Duke University Religion Index; OR = odds ratio; PR = prevalence ratio; OCD = obsessive-compulsive disorder; PTSD = post-traumatic stress disorder.

Characteristic	PR (95% CI)	p		
DUREL total score, per point	1.06 (1.03-1.09)	<0.001		
Sex: male vs. female	0.90 (0.80-1.01)	0.071		
Age 26-45 vs. 18-25	1.29 (1.02-1.62)	0.03		
Age >45 vs. 18-25	1.21 (0.96-1.54)	0.108		
Diagnosis: mood vs. anxiety/OCD/PTSD	0.96 (0.86-1.06)	0.385		
Diagnosis: psychotic vs. anxiety/OCD/PTSD	0.98 (0.82-1.15)	0.77		
Diagnosis: personality/other vs. anxiety/OCD/PTSD	0.69 (0.51-0.93)	0.014		
Prior discussion helpful vs. never discussed	0.99 (0.91-1.07)	0.764		
Prior discussion not helpful vs. never discussed	0.63 (0.35-1.12)	0.113		
Characteristic	Ordinal logistic OR (95% CI)	p	Linear β (95% CI)	p
DUREL total score, per point	1.35 (1.24-1.47)	<0.001	0.121 (0.092-0.151)	<0.001
Sex: male vs. female	0.89 (0.49-1.60)	0.691	−0.087 (−0.316 to 0.142)	0.453
Age 26-35 vs. 18-25	5.88 (2.53-13.69)	<0.001	0.791 (0.459-1.123)	<0.001
Age 36-45 vs. 18-25	4.47 (1.93-10.39)	<0.001	0.633 (0.293-0.972)	<0.001
Age 46-55 vs. 18-25	8.70 (3.17-23.92)	<0.001	0.842 (0.461-1.222)	<0.001
Age >55 vs. 18-25	2.50 (0.73-8.54)	0.144	0.451 (−0.011 to 0.913)	0.056

## Discussion

Psychiatry and religion have traditionally offered different perspectives on complex aspects of human experience, leading to a longstanding divide between the two fields. Growing evidence, however, suggests this gap has narrowed: religious and spiritual involvement is associated with lower depression and anxiety, greater longevity, and improved well-being [15-17], and the inclusion of a diagnostic category for religious and spiritual problems in DSM-IV acknowledged that such experiences are not inherently indicative of psychopathology [4]. This study measured psychiatric patients’ preference for discussing R/S during outpatient care at Al-Noor Specialist Hospital, Makkah, Saudi Arabia.

This study found that most psychiatric outpatients considered psychiatrist-initiated R/S discussion acceptable, although only 38.6% actively preferred it while 43.6% were open to it only if clinically relevant. Acceptability was associated with higher religiosity, specifically with intrinsic religiosity rather than with frequency of religious practice, and with older age, but not with sex or education. That intrinsic religiosity was the only domain independently associated with the outcome when all three were modeled together suggests that it is the personal salience of belief, rather than observable religious behavior, that shapes willingness to discuss religion with a psychiatrist. This distinction is clinically useful, because frequency of mosque attendance is more readily observed than internalized belief and would be a poor proxy for a patient’s willingness to have the subject raised.

Although previous studies primarily investigated psychiatrists' attitudes rather than patients' preferences, our findings align with evidence that psychiatrists generally recognize the clinical importance of R/S: Curlin et al. found 93% of psychiatrists considered it appropriate to inquire about patients' religious beliefs and 87% routinely did so [18], while Brazilian studies found 88.1% believed they should ask about R/S and roughly three-quarters considered it important to integrate into practice [19,20]. Collectively, these findings suggest concordance between patients' willingness to discuss R/S and psychiatrists' recognition of its relevance.

A key finding was that higher religiosity, across all DUREL domains, was independently associated with greater receptiveness to R/S discussion, with a clear dose-response pattern across religiosity tertiles. Similar patterns have been reported among psychiatrists themselves, where greater personal religiosity was associated with more frequent inquiry about patients' religious beliefs and greater perceived relevance of R/S to practice [19,20]. Although these studies evaluated physicians rather than patients, they support the notion that religiosity shapes attitudes toward incorporating R/S into mental healthcare. Similarly, studies among patients with schizophrenia found positive associations between spirituality and adaptive coping strategies, including positive reappraisal, seeking social support, self-control, and problem-solving [20], and an international study of schizophrenia and bipolar disorder found spiritual well-being to be the strongest predictor of psychological resilience [21]. These findings reinforce that religiosity and spirituality constitute meaningful psychological resources, which may explain why more religious patients in our study were more receptive to discussing these issues during psychiatric consultations.

Age was another significant predictor, with patients older than 25 years more likely to welcome clinician-initiated R/S discussions than younger adults. This differs from Das et al.'s schizophrenia study, which found no age association with overall spirituality, though younger patients used certain coping strategies more; the discrepancy may reflect our more heterogeneous outpatient sample, or that older adults place greater value on religious guidance when coping with mental illness [22]. Sex was not independently associated with receptiveness after adjustment, consistent with previous studies reporting no significant relationship between sex and religious/spiritual characteristics or clinicians' R/S approaches [20,21], suggesting openness to R/S discussion depends more on personal religiosity than demographic characteristics. Education also showed no independent association despite a borderline unadjusted association, consistent with prior studies [19,20]; although Bachelor's-level education was associated with slightly lower agreement in one secondary model, educational background appears to have limited influence compared with intrinsic religiosity.

In an exploratory model, a combined personality/other diagnostic category showed lower acceptability relative to anxiety, obsessive-compulsive, and trauma-related disorders (PR: 0.69, 95% CI: 0.51-0.93). Among the 18 respondents with borderline personality disorder, five (27.8%) were classified as receptive. These estimates derive from small subgroups within an exploratory model with a low events-per-variable ratio and wide confidence intervals, and no mechanism can be inferred from them. We report them for completeness and to inform sample-size planning in future work; they should not be used to draw conclusions about any diagnostic group, and replication in adequately powered samples is required before any interpretation is warranted. Future multicenter and longitudinal studies are warranted to confirm these findings, explore diagnosis-specific differences, including the receptiveness of patients with borderline personality disorder, and evaluate whether incorporating spiritual discussions into psychiatric care improves patient satisfaction, therapeutic alliance, treatment adherence, and mental health outcomes.

This study has several strengths. To our knowledge, it is among the first in Saudi Arabia to examine psychiatric patients' own preferences regarding clinician-initiated discussion of R/S, a literature that has otherwise focused on clinicians' attitudes. Diagnoses were assigned by treating psychiatrists using DSM-5 criteria rather than self-reported, the response rate was 78.0%, and the DUREL domains were analyzed separately as the instrument developers recommend, allowing internalized belief to be distinguished from religious practice.

Several limitations should be considered. The primary outcome was derived from non-validated, study-specific items, and the prevalence estimate proved sensitive to how the outcome was defined, ranging from 38.6% to 88.6% across four prespecified definitions; no single figure should be quoted without that qualification. No formally validated Arabic version of the DUREL has been published to our knowledge, and the version used here was a study-specific forward translation whose structural validity in this population is unestablished.

Exposure and outcome were measured within the same instrument at the same sitting, so the association may be inflated by common-method variance, and there is semantic overlap between the intrinsic religiosity items and endorsement of religiously framed discussion. Socially desirable responding is a particular concern given the centrality of religious observance in Saudi society and the administration of the questionnaire by psychiatrists within the clinic; patients who declined may also differ systematically from participants, so the observed prevalence may be an upper-bound estimate.

Finally, the cross-sectional design cannot establish temporality, and all estimates are associational. Participants were ambulatory, Arabic-literate outpatients at a single tertiary center, so the findings should not be extended to inpatients, patients in acute illness episodes, or other settings. The achieved sample fell short of the calculated target because recruitment was bounded by a fixed three-month window, and all diagnosis-specific findings derive from an exploratory model with sparse subgroups; these are hypothesis-generating only and require confirmation in larger, multicenter studies.

## Conclusions

In this single-center sample of Saudi psychiatric outpatients, most respondents indicated that they would find a psychiatrist-initiated question about religion or spirituality acceptable, and greater religiosity - particularly intrinsic religiosity - was associated with greater stated acceptability. These findings describe stated preferences at a single point in time; they do not establish the acceptability, feasibility, or clinical benefit of routine spiritual assessment, which this study did not evaluate. They support an individualized approach in which clinicians seek permission before raising religion or spirituality and respect the preferences of the substantial minority who prefer no discussion (9.9%) or who prefer to raise the topic themselves (6.4%). Notably, 18.3% of respondents considered such discussion appropriate only if they themselves initiated it, and among non-receptive respondents, the most frequently endorsed reasons were a wish to keep R/S private and fear of being misunderstood or judged - concerns that argue against routine, unsolicited inquiry. Whether permission-based inquiry improves therapeutic alliance, satisfaction, or clinical outcomes requires prospective study.

## Appendices

Study questionnaire

Section A: Sociodemographic Characteristics

• Sex: Male / Female

• Age group: 18-25 / 26-35 / 36-45 / 46-55 / >55 years

• Marital status: Single / Married / Divorced / Widowed

• Educational level: Less than secondary school / Secondary school / Diploma / Bachelor’s degree / Postgraduate

Section B: Clinical Characteristics

• Psychiatric diagnosis:

• Duration of psychiatric follow-up:

Section C: Preferences Regarding Discussion of Religion and Spirituality

• Would you prefer your psychiatrist to ask you a brief, neutral question about whether religion or spirituality is relevant to your care?

• If you prefer not to discuss religion or spirituality with your psychiatrist, what are your reasons?

• If you consider religion or spirituality relevant to your condition, would you prefer to discuss it during the consultation?

• When would you consider it appropriate for a psychiatrist to discuss religion or spirituality?

Section D: Religiosity

The five-item Duke University Religion Index (DUREL) [12].

Section E: Previous Experience

• Have you previously discussed religion or spirituality with a psychiatrist?

• If yes, was the discussion helpful?
